# The Exploitation of pH-Responsive Eudragit-Coated Mesoporous Silica Nanostructures in the Repurposing of Terbinafine Hydrochloride for Targeted Colon Cancer Inhibition: Design Optimization, In Vitro Characterization, and Cytotoxicity Assessment

**DOI:** 10.3390/pharmaceutics15122677

**Published:** 2023-11-26

**Authors:** Mohammad H. Alyami, Abeer A. Musallam, Tarek M. Ibrahim, Mahmoud A. Mahdy, Hanan M. Elnahas, Reem A. Aldeeb

**Affiliations:** 1 Department of Pharmaceutics, College of Pharmacy, Najran University, Najran 66462, Saudi Arabia; 2 Department of Pharmaceutics, College of Pharmaceutical Sciences and Drug Manufacturing, Misr University for Science and Technology, 6th of October City 12582, Egypt; 3 Department of Pharmaceutics, Faculty of Pharmacy, Zagazig University, Zagazig 44519, Egypt

**Keywords:** terbinafine hydrochloride, drug repurposing, pH-responsive mesoporous silica nanostructures, Eudragit, DiSupLo technique, targeted therapy, colon cancer

## Abstract

Targeted drug delivery is achieving great success in cancer therapy due to its potential to deliver drugs directly to the action site. Terbinafine hydrochloride (TER) is a broad-spectrum anti-fungal drug that has been found to have some potential anti-tumor effects in the treatment of colon cancer. We aimed here to design and develop pH-sensitive Eudragit (Eud)-coated mesoporous silica nanostructures (MSNs) to control drug release in response to changes in pH. The diffusion-supported loading (DiSupLo) technique was applied for loading TER into the MSNs. The formulation was optimized by a D-optimal design, which permits the concurrent assessment of the influence of drug/MSN%, coat concentration, and MSN type on the drug entrapment efficiency (EE) and its release performance. The optimal formula displayed a high EE of 96.49%, minimizing the release in pH 1.2 to 16.15% and maximizing the release in pH 7.4 to 78.09%. The cytotoxicity of the optimal formula on the colon cancer cells HT-29 was higher than it was with TER alone by 2.8-fold. Apoptosis in cancer cells exposed to the optimum formula was boosted as compared to what it was with the plain TER by 1.2-fold and it was more efficient in arresting cells during the G0/G1 and S stages of the cell cycle. Accordingly, the repurposing of TER utilizing Eud/MSNs is a promising technique for targeted colon cancer therapy.

## 1. Introduction

Colon cancer is one of the most prevalent types of cancer in the world. It is the world’s second-greatest cause of cancer-related mortality. By 2040, the incidence of colon cancer is expected to grow to 3.2 million new cases per year (a 63% rise) and 1.6 million deaths annually (an increase of 73%) according to the statistics of the World Health Organization (WHO), 2023 [1]. The prognosis for colon cancer varies depending on the stage at diagnosis. Survival rates are generally higher for cancers that are detected in the early stages compared to those diagnosed at advanced stages. Surgical resection is an effective cure for early-stage cancer; however, unfortunately, more than half of the cases are identified at later stages, necessitating the use of chemotherapy as the standard treatment to extend survival [2].

Synthetically engineered anti-cancer medications are employed to eliminate cancerous cells by impeding their proliferation or arresting the process of cell division [3]. Although anti-cancer drugs are successful in treating cancer cells, they have the drawback of being toxic to healthy cells. This lack of specificity leads to significant side effects and makes them difficult for patients to tolerate [4]. These adverse effects greatly impact the overall quality of life and treatment effectiveness, particularly in older patients [2]. Scientists are currently exploring a novel approach that can specifically seek tumors. One highly promising technology is targeted drug delivery, which involves incorporating medications into nanocarriers like mesoporous silica nanostructures (MSNs), liposomes, nanoemulsions, or nanoparticles. This method offers several advantages including enhanced drug efficacy; reduced toxicity; and overcoming various obstacles, such as low drug solubility and dissolution rate, while also facilitating the precise delivery of drugs to specific cancer cells [4].

The MSNs, a subset of inorganic nanoparticles, are becoming more commonly employed in medication delivery because of their reduced cytotoxicity at high concentrations compared to other inorganic nanomaterials [5]. According to substantial toxicological, safety, and epidemiological research, these biodegradable and biocompatible chemicals pose no environmental or health risk [6]. In addition, the MSNs are able to entrap a large amount of medication and then release it in a controlled release manner, making them commonly used in the production of sustained release systems that increase the drug’s therapeutic effectiveness and decrease the adverse effects [7]. Furthermore, the amorphization of drug crystals and their increasing surface area by loading into MSNs lead to a boost in the drug solubility and dissolution rate [8]. Additionally, employing MSNs as a delivery method enables an efficient load release from the carrier at targeted sites using different external stimuli, such as temperature, electric charge, time, or light, as well as interior stimuli, such as pH or enzymes [9]. The coating of MSNs with pH-sensitive polymers, such as Eudragit (Eud), may cause drug release in response to pH stimuli, hence allowing for enhanced control of the drug release [10].

The pH-responsive excipients have been extensively employed in cancers and inflamed tissues to target the intracellular components [9]. They offer special benefits for colonic administration due to their characteristics of drug release at a specified pH and their compatibility with the physiological conditions of the gastrointestinal tract (GIT) [10]. Eudragit S100 and Eudragit RL-100 polymers are among the most studied pH-responsive polymers for the oral delivery of small molecules. Eudragit S100 is a commercialized pH-sensitive acrylate for the enteric coating of tablets and capsules. It has a dissociation pH above 7, which is suitable for colonic delivery [10,11]. Eudragit RL-100 is a copolymer of methacrylic acid and methyl methacrylate, with a relatively high content of quaternary ammonium groups. Eudragit RL-100 is known for its ability to remain intact in the acidic environment of the stomach and to release drugs in a controlled manner in the colon [12].

A variety of approaches have been utilized to embed active medicinal substances within the pores of MSNs. Drug loading into the pores of mesoporous silica may be performed by two procedures: methods that are solvent-free and methods that are solvent-based [13]. The diffusion-supported loading (DiSupLo) technique is a new exceptionally simple and highly effective technique for drugs to be loaded into the pores of MSNs. This approach has the advantages of the solvent-based methods while eliminating their drawbacks. As it is a fast process requiring a minimum number of solvents, resulting in environmental friendliness and economic justification, no specific equipment or difficult experimental conditions are required to carry out the prosses [14].

The process of developing a new drug in the pharmaceutical industry is a complex and lengthy undertaking. The Food and Drug Administration (FDA) outlines a four-stage process that includes discovery, development, preclinical research, and clinical trials before marketing the drug. The FDA considers all those stages to extend over a span of 12 to 15 years [15]. Drug repurposing, also known as drug repositioning, has proven to be a valuable strategy in addressing the time-consuming process of drug discovery. The concept of repurposing involves exploring alternative therapeutic applications for drugs that have already been approved for different uses. In other words, it involves finding new ways to utilize previously authorized medications [16,17].

Terbinafine hydrochloride (TER) is an anti-fungal agent that can be applied topically or taken orally. It works by inhibiting a specific enzyme called fungal squalene oxidase, which is involved in the production of ergosterols in fungi. This inhibition allows the squalene to accumulate within the fungal cells, causing their death [18]. TER has been found to have a favorable safety profile and minimal interactions with other drugs. Recent research has indicated that TER may possess anti-cancer properties for colon cancer inhibition as it can halt the growth of cancer cells during the G0/G1 phase [19].

This study aimed to prepare a novel surface-modified MSNs formulation by the DiSupLo technique for the repurposing of TER to target and inhibit tumorigenesis in human colon cancer (HT-29). The D-optimal design was embraced to examine the optimal conditions for loading TER into MSNs to obtain the optimal TER formula with the desired drug encapsulation and its colonic release features. Producing a low initial release in the acidic condition of the stomach and a prolonged residence in the colonic region, which is crucial for colon cancer therapy, was targeted. Additionally, the cytotoxicity of the optimal formula was determined by investigating the cell viability, apoptosis, and cell cycle arrest to assess the prepared optimized formula’s ability to produce programmed cell death.

## 2. Materials and Methods

### 2.1. Materials

TER was gratefully supplied by the Egyptian Group for Pharmaceutical Industries (Obour, Qalyubia, Egypt). MCM-41 and SBA-15 silica were obtained by the XFNANO Materials Tech Co., Ltd. (Xushuguan, Beijing, China). Eud RL-100 (catalog number: 3343-24-1) and S-100 (catalog number: 25086-15-1) were delivered by the Sigma-Aldrich Chemical Co. (St. Louis, MO, USA). Disodium hydrogen orthophosphate and potassium dihydrogen orthophosphate were received from the El-Nasr Pharmaceutical Chemicals Company (Oubour, Qalyubia, Egypt). Absolute ethanol (99%) was obtained from the El-Nasr Pharmaceutical Chemicals Company (Oubour, Qalyubia, Egypt). All chemicals and reagents were of analytical grade.

### 2.2. Methods

#### 2.2.1. Experimental Design

The D-optimal design was used to investigate the principle and interactive variable effects on the examined responses by using Design-Expert^®^ software, version 13 [20]. An initial screening of the independent factors as drug/MSN percentage (A), coat concentration percentage (B), and MSN type (C) was conducted to select the most effective of them on the chosen dependent factors. The dependent responses were entrapment efficiency percent (EE; Y_1_), percentage of TER released in pH 1.2 after 2 h (Q_2_ in pH 1.2; Y_2_), and percentage of TER released in pH 7.4 after 24 h (Q_24_ in pH 7.4; Y_3_). Table 1 illustrates the independent factors with targeted responses. Furthermore, Table 2 lists the design’s suggested twelve formulas and the resulting responses.

#### 2.2.2. Optimization of Formulation Components

After conducting the statistical analysis by one-way analysis of variance (ANOVA), an optimization technique was employed to optimize the formulation factors of the prepared formulas. Table 1 outlines the desired goals for each dependent response. A high desirability value suggests that the response is compatible with its desired value [6]. Following that, the responses of the optimized formulation were re-examined, and the comparison took place between the experimental values and predicted values suggested by the D-optimal design. Subsequently, the percentage of prediction error was calculated according to the following equation [21]:

(1) $$\mathrm{Prediction}\;\mathrm{error}\;(\%)=\frac{\mathrm{predicted}\;\mathrm{value}\;-\;\mathrm{experimental}\;\mathrm{value}\;}{\mathrm{predicted}\;\mathrm{value}}\times 100$$

#### 2.2.3. Loading of TER into MSNs

The DiSupLo technique was used for the TER loading procedure. In each formula, 90 mg of TER and MSNs with the desired ratios were physically mixed and homogenized. The mixture’s weight stayed unchanged during all examinations. The studied drug to MSN ratios were 25% *w*/*w*, 33.33% *w*/*w*, and 50% *w*/*w*. The solid mixture was placed in an open weighing container and then kept in a tightly sealed container containing 15 mL ethanol at 25 °C for 3 h. Lastly, ethanol was evaporated from the prepared formulas in a vacuum oven (Fisher Scientific Isotemp, Oven 100 series, Model 127 G, Hampton, VA, USA) heated to 50 °C for 6 h [13,14].

#### 2.2.4. Preparation of Eud-Coated TER-Loaded MSNs

The coating process of the prepared TER-MSNs was performed by the incipient wetness method. Eud S-100 was dissolved in 100 mL of ethanol to prepare various concentrations of 5% *w/v* and 10% *w*/*v*. The ethanolic solution of Eud RL-100 was also prepared by the same method at concentrations of 5% *w/v* and 10% *w*/*v*. After that, 50 mg of TER-loaded MSNs were coated by applying 1 mL of Eud S-100 followed by 1 mL of Eud RL-100, drop by drop. At 25 °C, the coated formulas were dried [22].

#### 2.2.5. Determination of EE

To conduct this test, 1 mg/mL of aqueous suspension of the prepared formulas was prepared and then centrifuged for 15 min at 15,000 rpm using a centrifuge (Sigma 3 K 30, Roedermark, Germany). The supernatant was sufficiently diluted by distilled water before being measured using a spectrophotometer (Shimadzu UV 1650 Spectrophotometer, Kyoto, Japan) at λ_max_ 282 nm [23]. The EE was determined according to the equation below:

(2) $$\mathrm{EE}\;(\%)=\frac{\mathrm{initial}\;\mathrm{amount}\;\mathrm{of}\;\mathrm{TER}\;-\;\mathrm{free}\;\mathrm{amount}\;\mathrm{of}\;\mathrm{TER}\;}{\mathrm{initial}\;\mathrm{amount}\;\mathrm{of}\;\mathrm{TER}}\times 100$$

#### 2.2.6. In Vitro Cumulative Release Study

The release behavior of plain TER and the formulas of the TER-MSNs and Eud/TER-MSNs were determined using the dialysis bag technique [24]. Prior to the experiment, the dialysis bags were soaked in each release medium overnight. The fixed amount of plain TER and formulas equivalent to 10 mg of TER were transferred to the dialysis bags (cut off 12–14 kDa) and tied at both ends. The bags were then placed in a beaker holding a dissolving media of 0.1N HCl (pH 1.2) as an indicative of gastric fluid for 2 h; then, a phosphate buffer saline (PBS) of pH 6.8 as an indicative of intestinal fluid for 4 h; followed by a mixture of PBS of pH 7.4 and ethanol (50:50) as an indicative of colonic fluid for 24 h [25]. The volume of each dissolving media was adjusted to attain the sink condition. The experiment involved suspending a sample in a receptor medium that had been preheated to 37 ± 0.5 °C. The suspension was shaken at 100 rpm in a thermostatic shaking water bath (Lab-Line Instruments, Melrose Park, IL, USA). At different time intervals, 3 mL of the receptor media was removed, filtered through a 0.45 µm filter, and reintroduced with an equivalent amount of medium to keep a consistent volume. Each sample was then determined for drug content using UV spectrophotometry at a wavelength of 282 nm. All experiments were conducted in triplicate and the release percentage was mentioned as the mean ± standard deviation (SD). The drug release percentage was estimated according to the equation below:

(3) $$\mathrm{Drug}\;\mathrm{released}\;(\%)=\frac{\mathrm{amount}\;\mathrm{of}\;\mathrm{TER}\;\mathrm{in}\;\mathrm{the}\;\mathrm{release}\;\mathrm{medium}\;}{\mathrm{amount}\;\mathrm{of}\;\mathrm{TER}\;\mathrm{loaded}\;\mathrm{into}\;\mathrm{MSNs}}\times 100$$

#### 2.2.7. In Vitro Characterization of the Optimal Formula

##### Kinetic Study of the Release Data

The release kinetics data were analyzed using several models: the zero-order model (Q_t_ = K_o_ · t), the first-order model (log Q_t_ = log Q_o_ − K · t/2.303), the Higuchi release model (Q_t_ = K_H_ · t^0.5^), the Korsmeyer–Peppas model (Q_t_/Q_∞_ = K_k_ · t^n^), and the Hixson–Crowell model (Q_o_^1/3^ − Q_t_^1/3^ = K_s_ · t). In these models, Q_t_ represents the amount of drug released; t is the time interval; Q_o_ is the initial amount of drug; Q_∞_ is the amount of drug released at infinity (∞); n is the release exponent; and K_o_, K, K_H_, K_s_, and K_k_ are the release rate constants corresponding to each model, respectively. The correlation coefficients (R^2^) were used to determine the drug release order [26].

##### Measurement of Particle Size (PS), Polydispersity Index (PDI), and Zeta Potential (ZP)

The particle size was evaluated by the technology of dynamic light scattering (DLS) by using a Zetasizer (Malvern, Nano–ZS90, Malvern, UK). Before measurement, an aqueous suspension of each sample was prepared and diluted appropriately at room temperature. Additionally, the PDI values were monitored to evaluate the sample size homogeneity. The zeta potential values of each sample were determined by inserting the materials into a transparent disposable zeta cell.

##### Gas Adsorption Manometry

To investigate the porosity of the MSNs and determine how the loaded drug affects it, nitrogen adsorption and desorption analyses were conducted. These analyses provided data on the surface characteristics of the tested formulas. The measurements were carried out at 77 K using a N_2_ adsorption/desorption analyzer (Quantachrome Instruments, NOVA touch 2LX, Boynton Beach, FL, USA).

##### Fourier-Transform Infrared (FT-IR) Spectroscopy

The FT-IR analysis was carried out to verify the effective TER loading and successful coating with Eud. The measurements were conducted using a FT-IR instrument (Shimadzu, 8400 S, Kyoto, Japan) with a KBr disk at a resolution of 4 cm^−1^ within the frequency range of 500–4000 cm^−1^.

##### Differential Scanning Calorimetry (DSC)

The DSC analysis was performed using a DSC instrument connected to a TA-501 thermal analyzer (Shimadzu, DSC-50, Tokyo, Japan). Each 5 mg sample was heated in a closed aluminum pan at a rate of 10 °C/min under a nitrogen flow of 20 mL/min at a temperature ranging from 25 to 250 °C.

##### Polarized Light Microscopy (PLM)

To assess the morphology of the examined formulas, PLM was employed. To obtain the pictures, the samples were viewed using a polarized light microscope (Olympus-SC180, Tokyo, Japan) under constant illumination conditions.

##### Transmission Electron Microscopy (TEM)

Detailed images of the morphology were obtained using TEM. Each formula was diluted and sonicated for 10 min. After that, on a copper grid, a drop of the sonicated formulas was then deposited and left to dry. Once dried, the sample was assessed using a TEM instrument (Jeol, JEM-2100, Tokyo, Japan) operating at 80 kV.

#### 2.2.8. Cytotoxicity Evaluation of the Optimal Formula

##### Cell Culture

This study utilizes human epithelial cells derived from the large intestine (FHC) as a normal human cell line and a human colon cell line (HT-29) as a cancer cell line. The cell lines were received from the American Type Culture Collection and grown in Dulbecco’s Modified Eagle Medium (DMEM) with 10% fetal bovine serum (FBS), 10 ug/mL of insulin, and 1% penicillin-streptomycin. All chemicals and reagents used in this study were sourced from Sigma (St. Louis, MO, USA).

##### In Vitro Cell Viability Assay

To assess the cytotoxicity of the examined formulas in colorectal cancer (HT-29), the 3-(4,5-dimethylthiazole-2-yl)-2,5-di-phenyl tetrazolium bromide (MTT) assay was employed. The experimental procedure involved seeding plate cells at a density of 1.2–1.8 × 10,000 cells/well in a 96-well plate with 100 µL of full growth media and 100 µL of the evaluated formulas per well. After incubating for 24 h, 100 µL of MTT was added and incubated for 4 h. Subsequently, to solubilize the formazan crystals, 1 mL of MTT dissolving solution was added, resulting in a purple color [27].

##### Apoptosis Assay by Flow Cytometry

To examine the processes of apoptosis and necrosis, the human colon cancer cell line (HT-29) was stained with an annexin V and propidium iodide (PI) kit at a cell density of 1 × 10^5^ cells/mL following the methods described in previous studies [28]. The samples were applied and incubated with the cells for 24 h. Staining was conducted according to the manufacturer’s instructions using the annexin-V FITC apoptosis detection kit from Sigma-Aldrich (St. Louis, MO, USA). Beckton Dickinson (BD) FACSCalibur™ flow cytometer (San Hose, CA, USA) was used to acquire and evaluate the data.

##### Cell Cycle Study

The impact of the tested formulas on the cell cycle of HT-29 cells was evaluated using the BD FACSCalibur™ flow cytometer. The stages of the cell cycle (G0/G1, S, and G2/M stage) were identified by measuring the absorption of PI through fluorescence-activated cell sorting (FACS) in HT-29 cells at a density of 1 × 10^5^ cells/mL. The samples were applied to the cells for 24 h before being analyzed for DNA content using the BD FACSCalibur™ flow cytometer.

#### 2.2.9. Statistical Analysis

The average ± SD was used to present the results and the statistical analysis was performed using the Student’s *t*-test and one-way ANOVA followed by Tukey’s post hoc test (n = 3) to determine the significance (*p* < 0.05). The analysis was conducted using SPSS software, version 20.

## 3. Results and Discussion

### 3.1. Preparation of Eud/TER-MSNs

In our study, the DiSupLo method is a very effective technique for TER being loaded into the pores of MSNs. Since TER dissolves readily in ethanol, the DiSuLo technique was employed, with ethanol vapor acting as the loading solvent, enabling TER molecules to penetrate the MSN pores effectively, evenly, and in a reasonable time [29]. The starting material in this method is a homogenized physical combination of TER and MSNs, which is housed in a closed container containing ethanol. The solid mixture and the solvent are not directly contacted; only the ethanol vapor and the solid matter are interacting. The ethanol vapor permeates the whole volume of the solid mixture, condenses locally, dissolves the drug in a very small amount of solvent, and then transfers the drug to the pores of the MSNs (Figure S1 Supplementary Material) [13]. To prepare the coated formula, the coating process was performed by a double coat of Eud S-100 and Eud RL-100. The process was performed by the incipient wetness method, which allows effective coating via the uniform distribution of coating material on the substrate. This is because the substrate was saturated with a solution of the coating material until it reached its maximum absorption capacity (Figure S1 Supplementary Material) [9].

### 3.2. Experimental Analysis

The D-optimal design was permitted for the assessment of the independent variables and the optimization and evaluation of their effects on the dependent variables (Table 1). The data were then analyzed using ANOVA through the Design Expert software, version 13, which provided regression equations and correlation coefficients. The findings clearly demonstrate that the selected independent factors were strongly correlated with the dependent variables, as indicated by the significant *p*-values (Table 3)

#### 3.2.1. Variables’ Influence on the EE (Y_1_)

**Figure 1:**
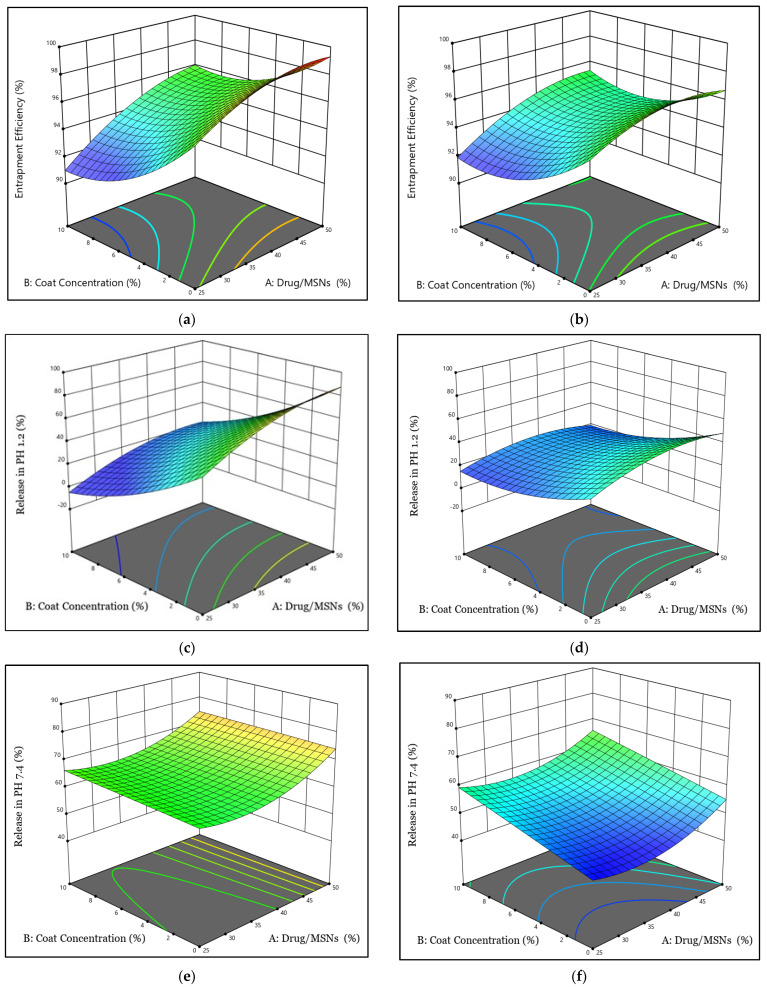
3D plots for the influence of factors A and B on (**a**) Y_1_ response using SBA-15, (**b**) Y_1_ response using MCM-41, (**c**) Y_2_ response using SBA-15, (**d**) Y_2_ response using MCM-41, (**e**) Y_3_ response using SBA-15, and (**f**) Y_3_ response using MCM-41.

In regards to the statistical analysis of EE (Table 3), the quadratic model was identified as a significant model because of its significant *p*-value (0.0001). This model was determined to have a high R^2^ (0.9924), indicating that it can identify around 99% of the deviations [21]. In this model, the appropriate precision value was (37.76), suggesting that the model could negotiate the design space. The EE of all formulae ranged from 91.94% (F7) to 99.53% (F1) (Table 2). The response 3D plot (Figure 1) and polynomial Equation (4) were used to examine the influence of interactive variables on EE, as shown below:

Y_1_ = + 94.78 + 1.16 A − 1.68 B + 0.41 C + 0.52 AB + 0.39 AC − 0.47 BC − 0.97 A^2^ + 1.45 B^2^ (4)

Regarding the above polynomial equation, factor (A) positively influenced the EE; as the drug proportion increased, the drug entrapment dramatically increased. The maximum EE can be observed when TER is loaded into MSNs at 50%; meanwhile, those loaded with TER at 25% display the lowest EE. This might be ascribed to the capability of MSN pores to encapsulate larger amounts of TER along with increasing the drug percentage [30].

The considerable drug entrapment into the pores of MSNs was contributed to the strong ionic interaction between the amine group of TER, which has a positive charge, and the negatively charged silanol group of MSNs [31]. However, the EE decreased after coating the TER-MSN with Eud due to the competition between the cationic Eud RL-100 and TER, resulting in some drug leaking in the coating solution [32].

Furthermore, the pore size of MSNs has a significant impact on the drug entrapment. SBA-15 has better loading efficiency than MCM-41. This might be because TER could distribute within the mesopores of SBA-15, which are larger and broader than MCM-41 pores, allowing TER to be entrapped into the pores easily and uniformly [6].

#### 3.2.2. Variables’ Influence on the Q_2_ in pH 1.2 (Y_2_)

The TER release profile from uncoated MSN formulas showed an initial burst release and then slow-release patterns. This could be observed in F1, F2, F3, and F4, as shown in Figure S2a,b Supplementary Material and Figure S3a,b Supplementary Material. This quick release occurred for the drug molecule placed close to the pore surface and near the dissolving fluid while the molecule entrapped deeper into the pores showed a slower release [33]. After coating the prepared formulas with Eud S-100 and Eud RL-100, the release process was significantly changed. Delaying and controlling the release pattern of TER from the coated formulas could be observed in F5, F6, F7, F8, F9, F10, F11, and F12 (Figures S2a,b and S3a,b Supplementary Material). For the targeted treatment of colon cancer diseases, this could be important for TER being delivered at a higher pH of colonic fluids and this could be achieved by using pH-sensitive Eud S-100. Additionally, coating with a second layer of Eud RL-100 was used to control the release in colonic environments. This could reduce the first burst release, often seen for drugs loaded into MSNs, and then release the loaded drug in a sustained release manner [22].

The ANOVA results presented in Table 3 indicate that the model achieved statistical significance, as evidenced by the significant *p*-value of 0.0002. Furthermore, the R^2^ value (0.94) was high. The adequate precision value was more than four (14.5), indicating the existence of a sufficient signal/noise ratio and the model’s ability to explore the design space. The TER release profile in pH 1.2 after 2 h varied between 12.59% (F7) and 98.65% (F1), as shown in Table 2 and Figure S2a,b. The surface 3D plot (Figure 1) and polynomial Equation (5) were utilized to assess the impact of the principal and interactive variables on the Y_2_ response:

Y_2_ = + 35.02 + 9.87 A − 21.45 B + 4.57 C − 2.17 AB + 5.74 AC − 8.95 BC − 8.42 A^2^ + 7.87 B^2^ (5)

In accordance with this equation, increasing the drug percentage had a positive effect on the release rate; whereas, increasing the proportion of MSNs to TER had the reverse effect. The large surface area of MSNs could enable more drug molecules to be loaded onto their surface, resulting in a rapid release rate at a high drug ratio. As the drug percentage increased, more drug molecules became accessible for release, resulting in a faster release rate [34]. Furthermore, greater and stronger interactions between the TER molecules and the mesoporous silica material could be obtained by increasing the drug percentage. This might lead to a more effective release of the drug from the silica matrix due to the massive amount of amorphous form of TER, which dissolved rapidly in the dissolving medium and showed a burst release [35].

The coating concentration was a detrimental parameter for the release of TER in pH 1.2, having a significant negative impact on the rate of TER being released from MSNs in the acidic condition. It was realized that the TER release rate was decreased by increasing the coating concentration from 5% to 10%; whereas, the non-coated formulas showed a burst TER release from the MSNs in the acidic pH and about 80% of the plain TER dissolved in the stomach pH, with a little amount having reached the colonic region. This was due to the pH-sensitive feature of Eud S-100, which prevented the fast dissolving of TER during the early passage of coated formulas through the stomach and upper small intestine. A significant delay of TER release was observed while increasing the polymer concentration [36].

The rate of TER release from MSNs was also affected by the silica pore size and pore structure [37]. The TER release rates from the formulas loaded into SBA-15 were higher than formulas loaded into MCM-41. This could be clarified due to SBA-15’s large pore size, which allows more dissolving fluid to enter the pore channels. This was effective in solubilizing and diffusing the drug molecules into the surrounding medium [38]. It might also be described by the SBA-15 pore structure, which allowed TER to quickly permeate the pore channel due to enough available space in the center of the pore [6].

#### 3.2.3. Variables’ Influence on the Q_24_ in pH 7.4 (Y_3_)

The release of TER from the MSNs was also evaluated and then submitted for computerized analysis to investigate the impacts of modifying the examined parameters on the formulations’ release percent values. According to the data in Table 3, the Y3 model has a significant *p*-value (0.0095), showing the model’s significance. The design software showed a high R^2^ of (0.85) and the adequate precision was larger than four, resulting in a reasonable signal/noise ratio and confirming the model’s capability in navigating the design space. The TER release profile after 24 h in pH 7.4 varies from 46.19% (F4) to 81.06% (F1), as shown in Table 2 and Figure S3a,b. The response 3D plot (Figure 1) and polynomial Equation (6) were used to demonstrate the effect of the main and interactive variables on the release rate:

Y_3_ = + 57.95 + 4.23 A + 3.35 B + 6.46 C − 0.56 AB + 0.48 AC − 2.54 BC + 5.36 A^2^ − 0.0976 B^2^ (6)

As indicated by this equation, increasing the drug percentage led to a rise in the TER release rate from the MSNs in pH 7.4. It was found that the TER release rate increased in the following order: 25% < 33.33% < 50%. As mentioned before, the rapid release rate at a high drug percentage was attributed to the massive amount of amorphous drug molecules, which dissolved quickly in the dissolving medium, hence bursting the TER release.

Furthermore, the coating concentration influenced the rate of TER release from MSNs in alkaline conditions. It was found that increasing the coating concentration in the following sequence enhanced the TER release rate (0% < 5% < 10%). This was attributed to the ability of the enteric Eud S-100 copolymer to dissolve at a pH of 7.4; therefore, the drug was seen to be released into the medium when the pH was raised above 7 [39]. This might be because the Eudragit S-100 polymer contains carboxyl groups, which ionize as the pH shifts from acidic to alkaline. Ionization happens at an alkaline pH disrupting the coat’s integrity and TER starts to leak from the MSNs [11]. In addition, TER was released from the coated formulas in a controlled manner, taking around 24 h to complete. The controllable release of TER might be attributable to the presence of the second layer of Eud RL-100, which demonstrated a sustained release of TER with no burst effect [40]. The hydrophobic property of the Eud RL-100 coat minimized the water penetration into the matrix, delaying the TER release process where the molecules diffused through the polymer coating and were then released slowly into the dissolving medium [41].

The release rate at pH 7.4 was also found to be affected by the silica pore size and pore geometry [37]. The TER release rates decreased when being loaded into MCM-41 more than they decreased with SBA-15, as previously stated, due to the SBA-15’s large pore size and the SBA-15 pore geometry [38].

### 3.3. Optimization and Validation of Variables

By employing Design-Expert^®^ software, version 13, the optimal TER-MSNs formula was selected based on the desirability criteria. Optimization targets were maximizing EE (Y_1_), minimizing the TER release in pH 1.2 after 2 h (Y_2_), and maximizing the TER release in pH 7.4 after 24 h (Y_3_) (Table 1). The suggested formula chosen by the software, which showed the highest desirability (0.793), was obtained by loading TER into SBA-15 where the drug percentage in the TER-MSNs mixture and the coat concentration were 50% and 10%, respectively (F10). The experimental and anticipated values were in a good correlation (Table 4), showing an acceptable prediction error percentage of less than 10% [42]. The optimization findings showed that the experimental design models were valid and applicable for assessing the influence of the tested variables on the evaluated responses.

### 3.4. In Vitro Characterization of Optimal Formula

#### 3.4.1. Kinetic Release Study

**Figure 2:**
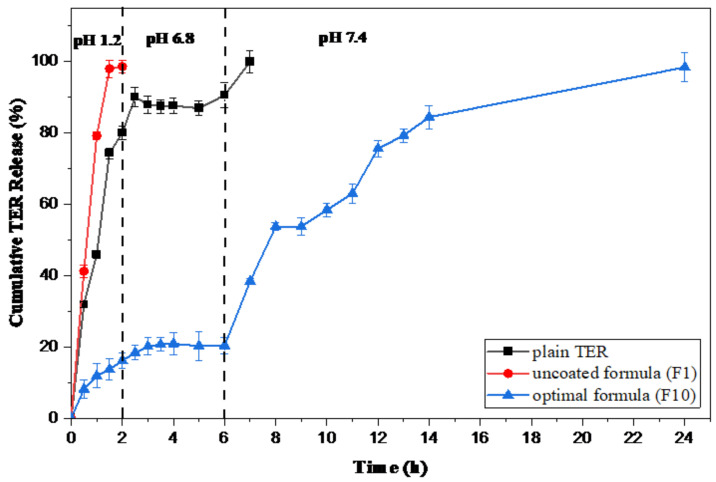
Cumulative release profiles of the optimal formula (F10) in comparison to uncoated F1 and plain TER.

A comparative study of the release profile of the optimized coated formula (F10) in different pHs of the GIT in comparison to the uncoated formula of the same composition (F1) and the plain TER was conducted (Figure 2). The drug release percentage of the optimal formula in pH 1.2 after an interval of 2 h was minimal (only about 16.15%). When the release study was carried out in PBS with a pH of 6.8 at an interval of 4 h, the release was increased from 16.15% to about 20%, showing a minimal amount of TER detected in the dissolving medium. When the pH was raised to 7.4, it was noticed that the drug was released into the medium and reached 78.09% after 24 h. The uncoated formula (F1) began to release TER, only after 30 min, in the release medium, in which just about all of the drug (98.65%) was released in pH 1.2 after 2 h. On the other hand, about 80% of plain TER dissolved in the acidic pH after 2 h, with 10% dissolved in the intestinal environment and the remaining amount released completely after 1 h of reaching the colonic environment.

By using the mathematical modeling equations, we evaluated the previous release data to determine the model that most properly describes their release patterns. A detailed examination of these samples demonstrated that the first-order equation provided the best match for their in vitro release data, showing that the release was dependent on drug concentration [39]. However, these release data are believed to follow the Hixson–Crowell model as the R2 values were higher than those of the Higuchi model (Table 5). According to the Hixson–Crowell model, the drug release is influenced mostly by the change in particle surface area, where the rate of drug release decreases over time with decreasing the drug surface area in response to dissolution or erosion [43]. In addition, according to the n values for each formula, the Korsemeyer–Peppas model could be employed to distinguish the following competing mechanisms: Fickian (diffusion-controlled), non-Fickian (anomalous), and case II transport (relaxation-controlled) [21]. The data in Table 5 Indicate that”the ’elease mechanisms of F1 and F10 follow a non-Fickian (anomalous) pattern. This suggests that both diffusion and swelling processes determine the drug release from these formulas. However, the plain TER release pattern fitted the Fickian diffusion mechanism.

#### 3.4.2. Measurement of PS, PDI, and ZP

The main physicochemical properties of the plain SBA-15, uncoated F1, and optimal formula (F10) are presented in Table 6. The average PS of the optimal formula was 176.1 ± 6.09 nm greater than that of SBA-15 and F1, indicating its successful coating with Eud RL-100 and S-100 [32]. The size of the nanoparticle in colorectal cancer treatment is critical. Nanoparticles with diameters of 100–200 nm showed a better effect than the bigger particles in terms of cancer targeting [44]. This might be attributed to the obvious enhanced permeability and increased retention in the epithelium leading to increasing the selective accumulation in the colon tissue [44]. The optimal formula’s PS distribution was examined and it revealed a narrow and uniform PS distribution with a PDI of 0.422 ± 0.06. The acceptable PDI range was reported to be from 0.05 to 0.7 and samples with PDI values out of this range would show unsuitable dynamic light scattering as they have a very broad particle size distribution [45]. The zeta potential of coated optimized F10 was found to be −15.8 ± 2.72 mV. The zeta potential value of the uncoated F1 formula was found to be low and negatively charged (−20.2 ± 4.62 mV) due to the presence of silanol groups (-Si-OH) that can dissociate in aqueous solutions, producing a negative surface potential [46]. The decline in the negativity of the F10 ZP value was due to the coating with Eud RL-100 having a positively charged quaternary amino group [10,47].

#### 3.4.3. Gas Adsorption Manometry

**Figure 3:**
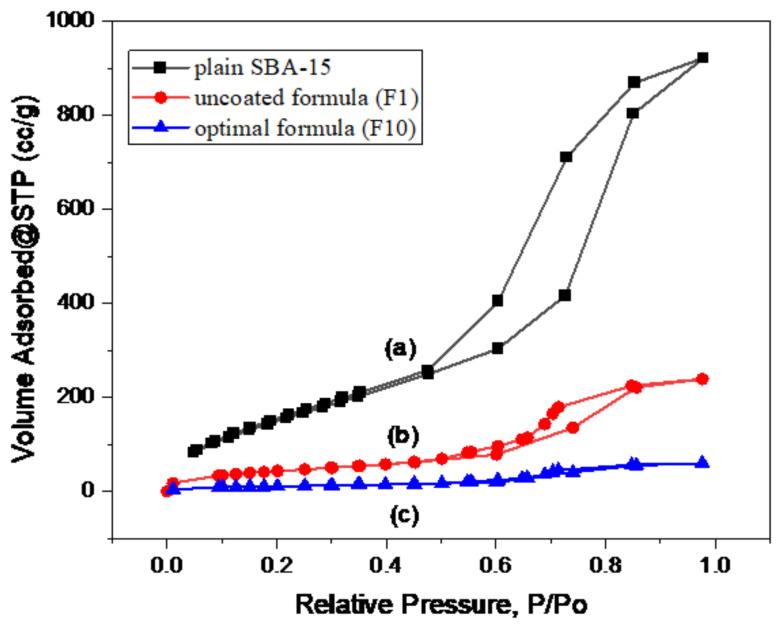
The isotherm curves of (a) plain SBA-15, (b) uncoated F1 formula, and (c) optimal F10 formula.

The N_2_ adsorption/desorption isotherms (Figure 3) demonstrated a typical type IV curve with an H1 hysteresis loop that was consistent with the cylinder-like pore channel of the plain SBA-15 [6]. In the uncoated formula (F1), the isotherm sequence remained without changes, indicating that the distinctive SBA-15 channel structure did not destroy after TER loading into the pores; whereas, the loaded samples’ hysteresis loop shut down at lower relative pressure levels than the plain SBA-15. This demonstrated that the pores were constricted to some extent due to the drug loading [48]. The surface area and pore volume of the uncoated formula (F1) decreased compared with plain SBA-15, as observed in Table 7. This reduction suggested that the DiSuLo approach effectively loaded TER into the SBA-15 pores. Whereas, after coating with Eud RL-100 in the optimal formula (F10), a great reduction in the surface characteristic could be observed.

#### 3.4.4. Fourier-Transform Infrared (FT-IR) Spectroscopy

**Figure 4:**
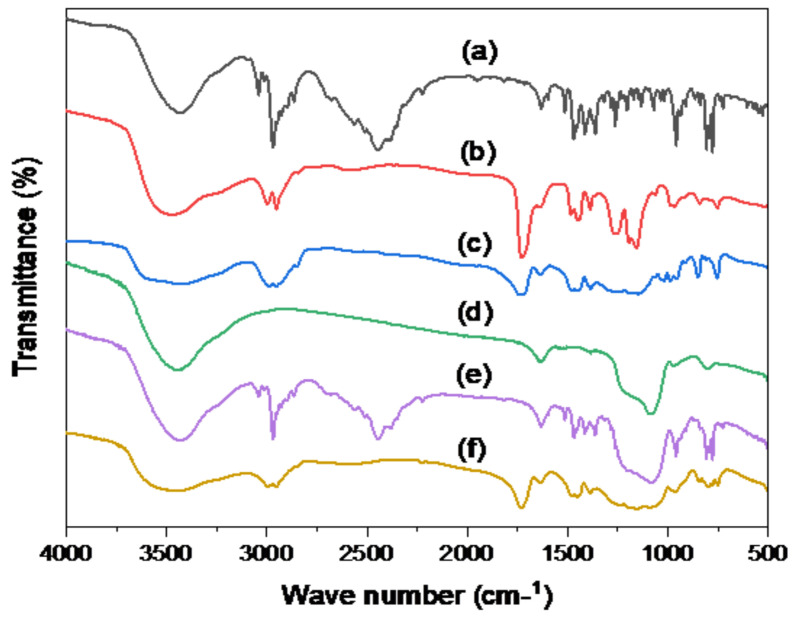
FT-IR of (a) plain TER, (b) Eud S-100, (c) Eud RL-100, (d) plain SBA-15, (e) uncoated F1, and (f) optimal F10 formula.

The FT-IR analysis was employed to evaluate drug/SBA-15 interactions based on the presence or lack of the distinctive functional groups [6]. As displayed in Figure 4a, plain TER revealed distinct peaks at 1408 cm^−1^ and 1384 cm^−1^ for the C-N and CH_3_ groups, respectively. The signal at 2983 cm^−1^ was ascribed to the aliphatic C-H group stretching; whereas, the peak at 3043 cm^−1^ suggested aromatic C-H groups. In addition, TER has a characteristic peak at about 3400 cm^−1^ for OH and NH groups [49]. In Figure 4b, Eud S-100 showed the sharp characteristic peak at 1750 cm^−1^, indicating the stretching vibration of the C=O of the ester groups. Peaks in the range of 2800–3000 cm^−1^ were correlated with C-H stretching vibrations of the methyl and methylene groups in the polymer backbone. In addition, peaks in the range of 1050–1250 cm^−1^ were associated with C-O stretching vibrations of the ester groups [12]. Figure 4C shows the FT-IR spectra obtained by the Eud RL100. The large bands in the spectra between 1190 cm^−1^ and 1250 cm^−1^ were attributable to the stretch of carbonyl (ester) groups. At 1734.01 cm^−1^, there were additional stretching bands of C=O ester vibration. The CH vibration may be identified at 1388.22, 1449.97, 2953, and 2992.11 cm^−1^ while the peak may be at 3437.91 cm^−1^, resulting from OH stretching [41]. In Figure 4d, SBA-15 has a broad distinctive peak of about 3439 cm^−1^ and a weak peak of around 970 cm^−1^, which reflected Si-OH stretching and bending vibrations. The carboxyl group (C-O-C) stretching vibration was assigned to the band at 1627 cm^−1^. The broad band at 1080 cm^−1^ might be caused by asymmetrical Si-O-Si stretching vibrations that overlap with Si-O-C, C-O-C, and Si-C bond vibrations. At around 804 cm^−1^, symmetrical stretching vibrations of Si-O-Si bonds belonging to ring structures were found. The bands in the range 468–449 cm^−1^ might be ascribed to Si-O-Si bond-bending vibrations [48,50]. The uncoated formula F1 (Figure 4e) shows the characteristic peaks of plain TER at 1408 cm^−1^, 1384 cm^−1^, 2983 cm^−1^, and 3043 cm^−1^, with the broad band around at the 1080 cm^−1^ peaks of plain SBA-15. This showed that the drug was present inside the formulation without any considerable interaction [51]. On the other hand, all these characteristic peaks disappeared in the optimized formula (F10) (Figure 4f). When the formula is coated with Eud, the sharp peaks at 1750 cm^−1^, peaks in the range of 2800–3000 cm^−1^ from Eud S-100, peaks in the region of 1190–1250 cm^−1^, peaks at 2953 cm^−1^, and peaks at 2992 cm^−1^ originating from Eud RL-100 could be observed. This would indicate the excellent coating of the optimized formula with Eud [7].

#### 3.4.5. Differential Scanning Calorimetry (DSC) Study

**Figure 5:**
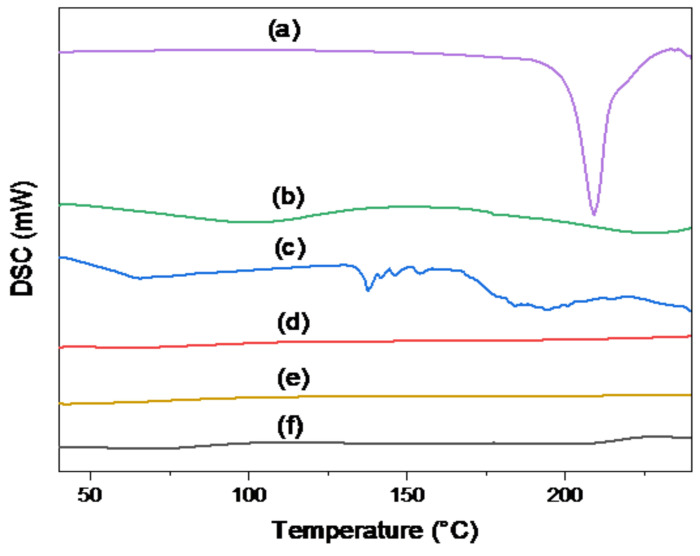
DSC curves of (a) plain TER, (b) Eud S-100, (c) Eud RL-100, (d) plain SBA-15, (e) uncoated F1 formula, and (f) optimal F10 formula.

To evaluate the entrapment of TER into the SBA-15 in the amorphous form, the DSC study was performed. The crystalline structure of TER was shown by a significant endothermic peak at 215 °C in the DSC spectra of TER (Figure 5a). The curves of Eud S-100 and Eud RL-100 in Figure 5b and Figure 5c, respectively, showed no characteristic peak, revealing that these polymers had an amorphous nature. In addition, the curve of plain SBA-15 also revealed the disappearance of any peaks (Figure 5d). On the other hand, the uncoated F1 formula (Figure 5e) and the optimal F10 formula (Figure 5f) exhibited no indication of the TER crystalline structure [52].

#### 3.4.6. Polarized Light Microscopy (PLM) Study

**Figure 6:**
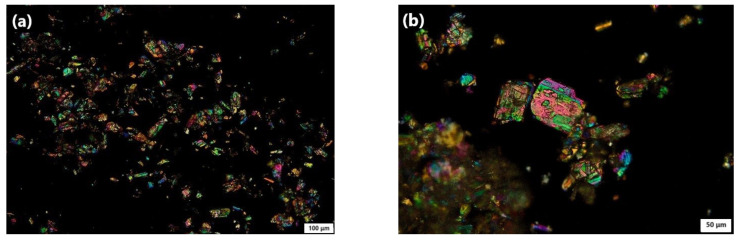

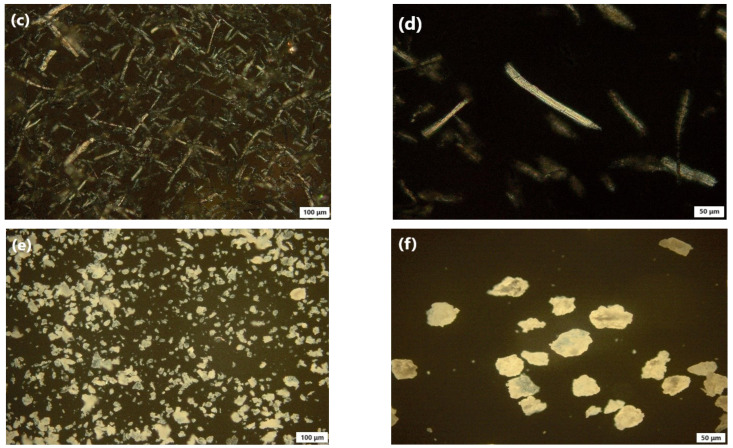
PLM images of (**a**,**b**) plain TER; scale bar 100 μm and 50 μm, respectively, (**c**,**d**) uncoated F1; scale bar 100 μm and 50 μm, respectively, and (**e**,**f**) optimized F10; scale bar 100 μm and 50 μm, respectively.

The morphology of the plain TER, uncoated F1, and the optimal formula (F10) could be observed by PLM images (Figure 6). The plain TER (Figure 6a,b) revealed brightly colorful crystals when observed using a polarized light microscope [53,54]. After loading TER into SBA-15 (Figure 6c,d), the image showed a broad distribution of sizes of typical rod-shaped particles while the drug crystals vanished upon loading TER into SBA-15, indicating its amorphous form [6]. After coating with Eud (RL100 and S-100) (Figure 6e,f), the coatings tended to form spherical or near-spherical shapes when applied to nanoparticles since the polymer tends to form a uniform and continuous coat around the nanoparticles resulting in a rounded shape with a smooth bright surface [41].

#### 3.4.7. Transmission Electron Microscopy (TEM) Study

**Figure 7:**
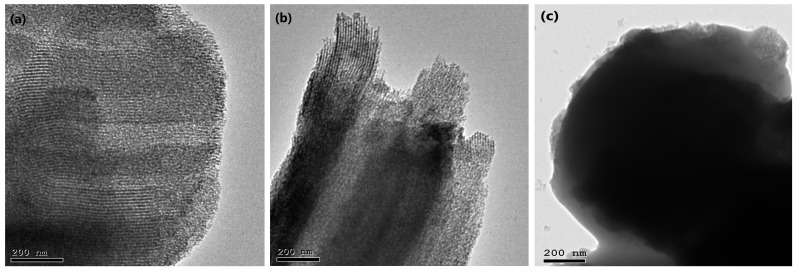
TEM images of (**a**) plain SBA-15, (**b**) uncoated F1, and (**c**) optimized coated F10.

The surface morphology of the uncoated formula (F1) and optimal formula (F10) can be examined by TEM images (Figure 7). The TEM image of uncoated F1 (Figure 7b) demonstrated that the formula was in the form of a short rod-like structure with an internal mesoporous structure in a mostly organized arrangement similar to the plain SBA-15 materials (Figure 7a). This indicated that the TER was effectively loaded into SBA-15 without pore destruction [48]. In Figure 7c, after the formula had been coated with Eud (RL100 and S-100), the surface showed a layer of covering substance, demonstrating the successful development of a thin layer of Eud around the nanostructure [55].

### 3.5. Cytotoxicity Evaluation of the Optimal Formula

#### 3.5.1. Cell Viability Study

**Figure 8:**
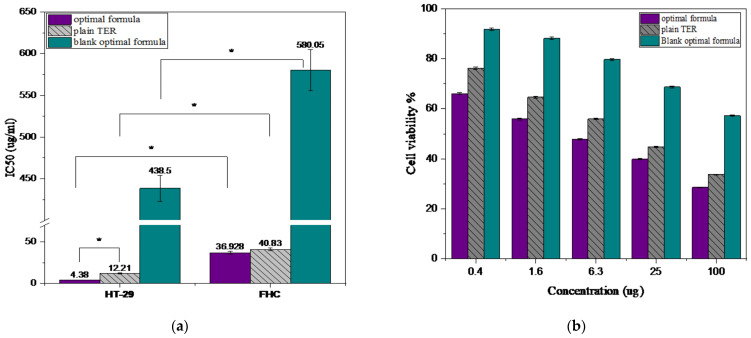
Cytotoxicity cell study: (**a**) IC50 of the uncoated optimal formula, plain TER, and blank optimal formula on the HT-29 cell line and FHC cell line and (**b**) the cell viability on HT-29 cancer cells of the optimal formula, plain TER, and blank optimal formula. *: significant at *p* < 0.05.

The cytotoxic impact of the repurposed anti-fungal TER as an anti-cancer treatment must be evaluated in terms of both its cytotoxic efficiency against malignant cells and its safety to normal host cells [16]. The MTT assay is the most extensively used cytotoxicity test. We assessed the biosafety and/or cytotoxicity of plain TER, the prepared optimal formula, and the blank optimal formula (Eud RL100 and S-100/SBA-15) on the human epithelial cell of the large intestine (FHC), which was chosen as a normal human cell line, and a human colorectal adenocarcinoma (HT-29), used as a cancer cell line. After 24 h of treatment with different concentrations (0.4, 1.6, 6.3, 25, and 100 µg/mL) of the three tested samples, the cell viability was assessed and expressed in the growth inhibitory concentration (IC50) values shown in Figure 8a. Notably, a statistically significant distinction in IC50 was observed between normal and cancerous cells when subjected to equivalent dosages (*p* ≤ 0.05). Accordingly, the tested formulas exhibited higher toxicity to HT-29 cancer cells in comparison to FHC. As the cytotoxicity on the normal cell was minimal, the tested formulas could be used to study the cytotoxicity on the HT-29 cell line in a safe manner [16]. The results showed that the cell viability of the HT-29 cell line was reduced in a dose-dependent way in response to treatment by either the plain TER, optimal formula, or blank optimal formula, as presented in Figure 8b. The IC50 of the plain TER was found to be 12.21 μg/mL. On the contrary, the optimal formula reduced the IC50 value significantly (*p* < 0.05) to 4.38 μg/mL, compared to the plain drug, while the blank formula value showed a very significant increase in the IC50 value of 438.5 μg/mL (*p* < 0.05), compared to the other formulas. We found that the loading of TER into SBA-15 in the optimal formula enhanced the cytotoxicity of TER by about 2.8-fold more, against the HT-29 cell line, than the plain TER and this was attributable to the mesoporous structure of SBA-15, which aided the cellular permeation of the loaded drugs. Additionally, the nano formulations’ small size has exhibited a major role in cellular ingestion. The high surface area and pore volume of SBA-15 could provide more contact points for the drug–cell interactions, increasing the uptake of drugs by cancer cells and improving their cytotoxicity [56].

#### 3.5.2. Apoptosis Assay by Flow Cytometry

**Figure 9:**
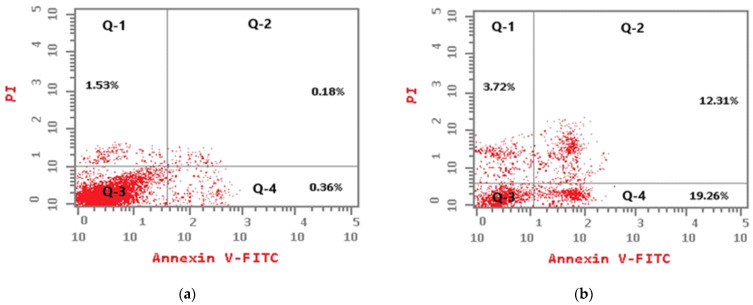

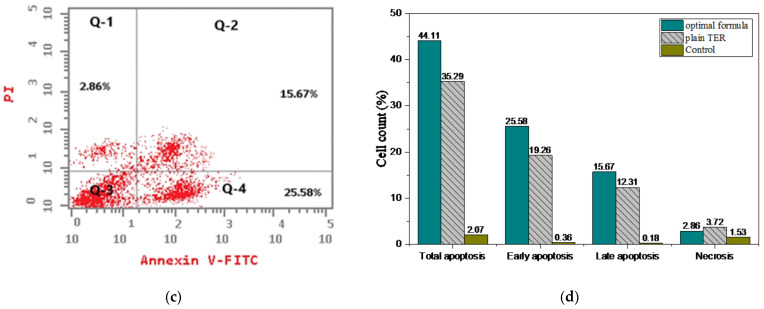
Apoptosis and necrosis assay by flow cytometry for (**a**) untreated cells, (**b**) plain TER, (**c**) optimal formula, and (**d**) cell count percentage analysis.

To investigate how TER and its nano-formulas could induce cytotoxic effects on the HT-29 cell line, we used flow cytometry to assess cell apoptosis using an Annexin V/PI test kit. As illustrated in Figure 9a–c, the test assessed early apoptosis (Q-4), late apoptosis (Q-2), necrosis (Q-1), and cell viability (Q-3). The test comprised untreated HT-29 cell lines (control) together with cells treated with plain TER and the optimal formula. Figure 9d demonstrates that both treatments increased total, early, and late apoptosis significantly compared to the control cells (*p* < 0.05). This was consistent with prior observations of the anti-tumor activity of TER [57]. Plain TER and the optimal formula demonstrated cytotoxicity because of the additive impact of early and late apoptosis but necrosis contributing to a lesser effect. According to the investigation, plain TER increased cellular apoptosis by approximately 17-fold more than the control. Meanwhile, when HT-29 cells were treated with optimal formula, the amount of apoptosis increased by about 21-fold in comparison with the control. Furthermore, the number of necrotic cells was reduced after treatment with the plain TER and the optimal formula in comparison with the control group while the latter exhibited quite a lower proportion of necrotic cells than the former. Moreover, the percentage of total apoptotic cells in the optimized formula was higher than in the plain TER by about 1.2-fold. Furthermore, the necrosis of the plain TER (3.72%) was greater than the optimized formula (2.86%) but in an insignificant manner. This finding proves the MSN’s potential to cause programmed cell death in colon cancer cells [58]. A cell cycle study was undertaken to understand more about the mechanism of the cytotoxicity of the produced compound.

#### 3.5.3. Cell Cycle Study

**Figure 10:**
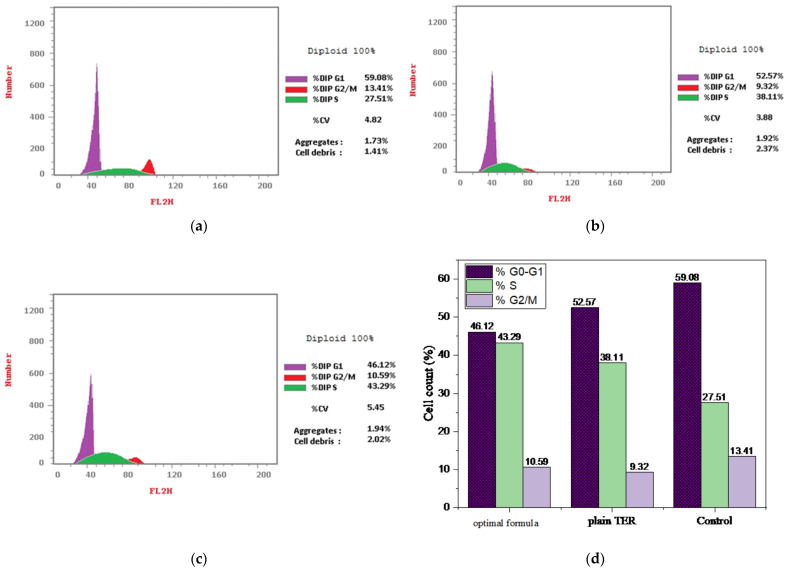
Cell cycle analyzed by flow cytometry for the (**a**) untreated HT-29 cells, (**b**) HT-29 treatment with plain TER, (**c**) HT-29 treatment with optimal formula, and (**d**) percentage of cell cycle analysis.

When the cell starts to divide into two cells, DNA must be replicated and then the nucleus is divided and cytoplasm partitioning happens. The cell cycle is primarily a process of cell reproduction through DNA replication [28]. Figure 10a–c shows the cell cycle behavior in the presence of TER and the optimal formula acting on HT-29 cells using nuclear PI staining and flow cytometry. The treatment of HT-29 cells with both plain TER and optimized MSNs resulted in a major accumulation of the cells in the G0-G1 phase, with 52.57% and 46.12% of cells accumulating in this phase, respectively, while boosting the proportion of cells in the S phase by 1.4 and 1.6 times, respectively, in comparison with the control HT-29 cells (Figure 10d). Furthermore, the G2/M phases of cells treated by plain TER and the optimal formula were reduced by 1.4 and 1.3, respectively, when compared to the control. This suggested that the optimal formula could primarily inhibit the colon cancer cells during the G0/G1 and S phases. This was crucial because the G0/G1 phase of the cell cycle was essential for preparing the cells for DNA replication and division while DNA synthesis occurs in the S phase [59]. Several drugs that target enzymes or proteins involved in DNA replication may disrupt this process. According to prior research conducted by He et al., 2021 [60], the TER’s cytotoxic effect on colon cancer was attributed to its ability to block the squalene epoxidase enzyme. It was previously discovered that squalene epoxidase can reduce cell apoptosis and accelerate cell cycle advancement in colon cancer cells while the inhibition of this enzyme might halt the colon cancer cells’ progression [61].

## 4. Conclusions

We satisfactorily produced and tested the pH-sensitive Eud-coated TER-MSNs as a promising colon cancer therapy. The high TER entrapment was achieved when the drug was loaded into SBA-15 and MCM-41 by utilization of the DiSupLo method. By using the D-optimal approach, the drug/MSN percentage (A) positively influenced the EE% (Y_1_), release in pH 1.2 after 2 h (Y_2_), and release in pH 7.4 after 24 h (Y_3_). Additionally, the coating concentration (B) was the detrimental parameter, having a negative impact on the EE (Y_1_) and the rate of TER release from MSNs in pH 1.2 (Y_2_) while positively impacting the release of TER in pH 7.4 (Y_3_). Furthermore, we found that loading TER into SBA-15 enhanced the three tested responses more than MCM-41. As a result, the optimal formula was achieved when TER was loaded into SBA-15 using a drug/MSN percentage of 50% and a coat concentration of 10% Eud RL-100 and RS-100. Throughout the physiochemical evaluation, TER was entrapped into the pores of SBA-15 in an amorphous form with no drug crystals to be observed. In addition, compared with TER alone, the prepared optimal formula showed a higher cytotoxic effect by about 2.8-fold and greater apoptotic cell death by about 1.2-fold in the HT-29 cell line; meanwhile, the cells are stopped in both the G0/G1 and S stages of the cell cycle. According to these findings, we propose that repurposing TER by loading it into MSNs coated with Eud might be an attractive technique for targeting colon cancer therapy.

## Figures and Tables

**Table 1:** D-optimal design with the independent variables and the examined responses.

Factors	Actual Levels (Coded)		
	Low Limit (−1)	Central Limit (0)	High Limit (+1)
A: Drug/MSN (%)	25	33.33	50
B: Coat concentration (%)	0	5	10
C: MSN type	MCM-41	SBA-15	
**Responses**	**Goals**		
Y_1_: EE (%)	Maximize		
Y_2_: Q_2_ in pH 1.2 (%)	Minimize		
Y_3_: Q_24_ in pH 7.4 (%)	Maximize		

Abbreviations: EE, entrapment efficiency percentage; Q_2_ in pH 1.2, percentage of TER released in pH 1.2 after 2 h; Q_24_ in pH 7.4, percentage of TER released in pH 7.4 after 24 h.

**Table 2:** Twelve experimental formulas suggested by a D-optimal design.

F	A: Drug/MSN (%)	B: Coat Concentration (%)	C: MSN Type	Y_1_: EE (%)	Y_2_: Q_2_ in pH 1.2 (%)	Y_3_: Q_24_ in pH 7.4 (%)
**1**	50	0	SBA-15	99.35 ± 3.16	98.65 ± 1.85	81.06 ± 2.96
**2**	33.33	0	SBA-15	98.34 ± 2.87	62.44 ± 2.21	57.80 ± 2.06
**3**	50	0	MCM-41	96.73 ± 0.88	44.87 ± 1.12	54.33 ± 2.21
**4**	25	0	MCM-41	95.28 ± 3.22	38.82 ± 2.21	46.19 ± 1.98
**5**	50	5	MCM-41	94.84 ± 1.67	30.27 ± 1.12	59.74 ± 2.37
**6**	33.33	5	MCM-41	93.97 ± 2.64	28.52 ± 2.21	56.45 ± 3.21
**7**	25	10	MCM-41	91.94 ± 4.65	12.59 ± 2.21	57.95 ± 2.06
**8**	50	5	SBA-15	95.81 ± 3.55	42.04 ± 3.11	65.99 ± 3.21
**9**	25	5	SBA-15	92.26 ± 2.33	15.68 ± 1.12	66.93 ± 3.06
**10**	50	10	SBA-15	96.49 ± 3.15	16.15 ± 2.21	78.09 ± 4.06
**11**	33.33	10	SBA-15	93.28 ± 1.26	19.79 ± 1.85	63.27 ± 3.06
**12**	50	10	MCM-41	95.24 ± 2.76	21.08 ± 2.21	66.16 ± 4.16

Abbreviations: EE, entrapment efficiency percentage; Q_2_ in pH 1.2, percentage of TER released in pH 1.2 after 2 h; Q_24_ in pH 7.4, percentage of TER released in pH 7.4 after 24 h.

**Table 3:** ANOVA results of the models for Y_1_–Y_3_ responses.

Responses	Model	R^2^	Adjusted R^2^	Predicted R^2^	Adequate Precision	*p*-Value	F-Ratio	Significance
Y_1_: EE (%)	Quadratic	0.99	0.98	0.94	37.76	0.0001	131.02	significant
Y_2_: Q_2_ in pH 1.2 (%)	Quadratic	0.94	0.89	0.54	14.5	0.0002	18.01	significant
Y_3_: Q_24_ in pH 7.4 (%)	Quadratic	0.85	0.71	−0.12	7.73	0.0095	6.13	significant

Abbreviations: EE, entrapment efficiency percentage; Q_2_ in pH 1.2, percentage of TER released in pH 1.2 after 2 h; Q_24_ in pH 7.4, percentage of TER released in pH 7.4 after 24 h; R^2^, correlation coefficient.

**Table 4:** Predicted and observed values of the optimal formula.

Factors	Optimized Level		
A: Drug/MSN (%)	50%		
B: Coat concentration (%)	10%		
C: Type of MSN	SBA-15		
**Responses**	**Observed**	**Predicted**	**Prediction error (%)**
Y_1_: EE (%)	96.49	96.05	−0.004
Y_2_: Q_2_ in pH 1.2 (%)	16.15	22.100	0.269
Y_3_: Q_24_ in pH 7.4 (%)	78.09	74.64	−0.046

Abbreviations: EE, entrapment efficiency percentage; Q_2_ in pH 1.2, percentage of TER released in pH 1.2 after 2 h; Q_24_ in pH 7.4, percentage of TER released in pH 7.4 after 24 h.

**Table 5:** Kinetic release study of plain TER, uncoated F1, and the optimal formula (F10).

Formulas	Zero Order Model	First Order Model	Higuchi Model	Hixson–Crowell Model	Korsmeyer–Peppas Model	
	R^2^	R^2^	R^2^	R^2^	R^2^	n
Plain TER	−0.0140	0.9605	0.7967	0.9320	0.9075	0.300
Uncoated F1	0.8594	0.9695	0.9665	0.9896	0.9687	0.554
Optimal coated F10	0.8931	0.9142	0.8369	0.9391	0.9315	0.791

Abbreviations: TER, terbinafine hydrochloride; R^2^, correlation coefficient.

**Table 6:** Measurement of the PS, PDI, and ZP of plain SBA-15, uncoated F1, and optimal F10.

Material	PS (nm)	PDI	ZP (mV)
Plain SBA-15	126.6 ± 3.40	0.476 ± 0.09	−23.7 ± 3.42
Uncoated F1	160.3 ± 4.43	0.420 ± 0.074	−20.2 ± 4.62
Optimal F10	176.1 ± 6.09	0.422 ± 0.06	−15.8 ± 2.72

Abbreviations: PS, particle size; PDI, polydispersity index; ZP, zeta potential.

**Table 7:** Surface characteristics of the plain TER, uncoated formula (F1), and optimal formula (F10).

Material	Specific Surface Area (m^2^/g)	Pore Volume (cc/g)
Plain SBA-15	621.13	1.42
Uncoated formula (F1)	156.58	0.42
Optimal formula (F10)	39.07	0.10

## Data Availability

The data presented in this study are available in this article and supplementary material.

## Supplementary Materials

The following supporting information can be downloaded at: https://www.mdpi.com/article/10.3390/pharmaceutics15122677/s1, Figure S1: graphical diagram of the Eud/TER- MSNs formulation. Figure S2: cumulative release profiles of the coated and uncoated formulas of (a) TER-loaded MSNs containing SBA-15 in pH 1.2 and (b) TER-loaded MSNs containing MCM-41 in pH 1.2 in comparison to plain TER. Figure S3: cumulative release profiles of the coated and uncoated formulas of (a) TER-loaded MSNs containing SBA-15 in pH 7.4 and (b) TER-loaded MSNs containing MCM-41 in pH 7.4.
