# Supplementary material for: The Exploitation of pH-Responsive Eudragit-Coated Mesoporous Silica Nanostructures in the Repurposing of Terbinafine Hydrochloride for Targeted Colon Cancer Inhibition: Design Optimization, In Vitro Characterization, and Cytotoxicity Assessment
Source: Pharmaceutics. 2023 Nov 26;15(12):2677. doi: 10.3390/pharmaceutics15122677 (PMC10747614; doi:10.3390/pharmaceutics15122677)
Supplement: Supplementary file 1 [file pharmaceutics-15-02677-s001.zip › pharmaceutics-2694745-supplementary.pdf]

Supplementary Material

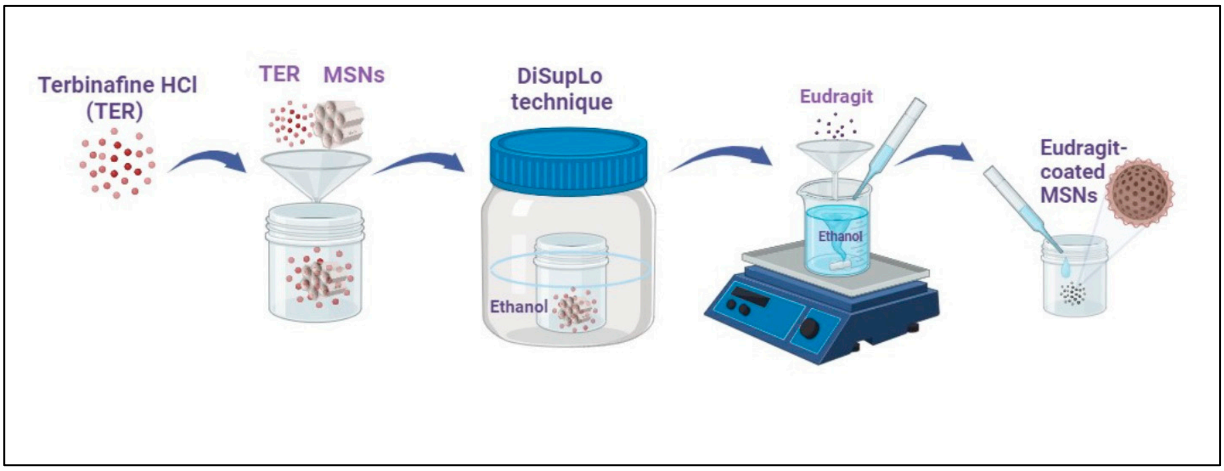

Figure S1. Graphical diagram of the Eud/TER- MSNs formulation.

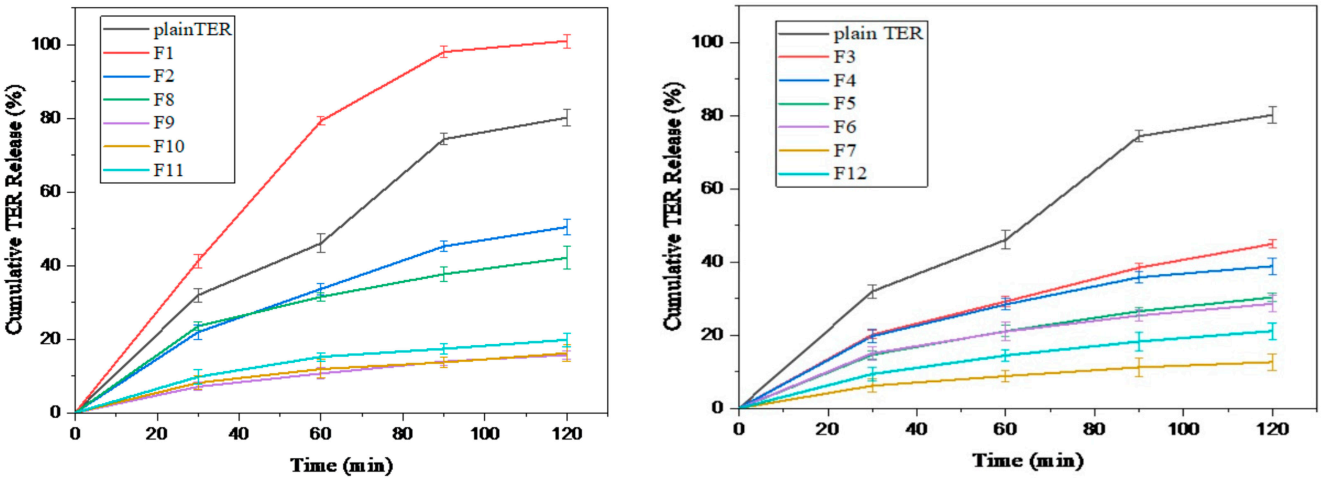

(a) (b)

Figure S2. Cumulative release profiles of the coated and uncoated formulas of (a) TER-loaded MSNs containing SBA-15 in pH 1.2 (b) TER-loaded MSNs containing MCM-41 in pH 1.2 in comparison to plain TER.

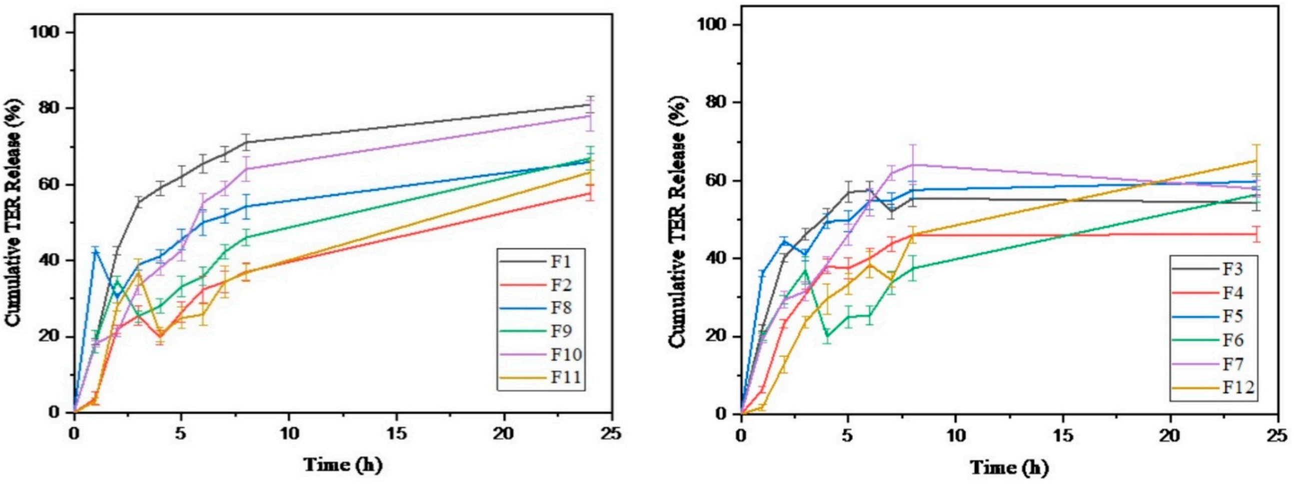

(a) (b)

Figure S3. Cumulative release profiles of the coated and uncoated formulas of (a) TER-loaded MSNs containing SBA-15 in pH 7.4 (b) TER-loaded MSNs containing MCM-41 in pH 7.4.
